# Magnetic Resonance Imaging Simulation of the Skull Base, Oral Cavity, Pharynx, and Larynx: Advanced Imaging Protocol for a Radiation Oncology Center

**DOI:** 10.64898/2026.10.05.26364781

**Published:** 2026-10-07

**Authors:** Natalie A. West, Lucas McCullum, Johnny T. Cheruvelil, Vi T. Dinh, Yao Ding, Jihong Wang, Komal Shah, Kim Learned, Melissa Chen, Jack Phan, Anna Lee, Adam Garden, David Rosenthal, Clifton D. Fuller

## Abstract

Head and neck radiation oncology, with its complex locality and epidemiology, requires various imaging techniques to achieve sufficient visualization. Through collaboration with medical physicists, radiation therapists, radiation oncologists, and neuroradiologists, our group clinically implemented a standard set of magnetic resonance imaging (MRI) sequences on an MR-simulation scanner for three anatomical regions: skull base, oral cavity, and larynx/pharynx. A total of 41 patients with head and neck cancer and 3 volunteers were scanned on either a 1.5T or 3T MR-simulation scanner. Sequences described include Dixon-based approaches, SPAIR-based approaches, short T2-based approaches, and quantitative imaging methods. We describe and outline the steps to optimize these notable MRI sequences to aid in successful implementation in a radiation oncology clinic.

## Full Text Availability

The license terms selected by the author(s) for this preprint version do not permit archiving in PMC. The full text is available from the preprint server.

